# Feasibility of a Tai Chi with Thera-Band Training Program: A Pilot Study

**DOI:** 10.3390/ijerph17228462

**Published:** 2020-11-16

**Authors:** Meiling Qi, Wendy Moyle, Cindy Jones, Benjamin Weeks

**Affiliations:** 1 School of Nursing, Shandong University, Jinan 250012, China; 2 Nathan Campus, Menzies Health Institute Queensland, Griffith University, Brisbane, QLD 4111, Australia; w.moyle@griffith.edu.au (W.M.); cjones@bond.edu.au (C.J.); b.weeks@griffith.edu.au (B.W.); 3 Nathan Campus, School of Nursing and Midwifery, Griffith University, Brisbane, QLD 4111, Australia; 4 Faculty of Health Sciences & Medicine, Bond University, Gold Coast, QLD 4226, Australia; 5 Gold Coast Campus, School of Allied Health Sciences, Griffith University, Gold Coast, QLD 4222, Australia

**Keywords:** strength training, Tai Chi, feasibility, physical fitness, psychological well-being, older sedentary office workers

## Abstract

Tai Chi, combined with Thera-band (TCTB) exercise may be associated with an improvement in health where it increases physical fitness, improves psychological well-being, and decreases pain. This paper aimed to determine the feasibility of TCTB exercise in older sedentary office workers. Forty office workers aged over 55 years participated in a pilot randomized controlled trial (i.e., 12-week TCTB exercise or Tai Chi exercise only). Feasibility of the TCTB exercise approach was ascertained through the recruitment and enrolment rate, acceptability of the study intervention by participants including retention and adherence rates, participants’ learning process, the appropriateness of data collection as well as the participants’ evaluation of the intervention. Recruitment took longer than planned, with a low recruitment rate of 2.0% (42/2020), but a high enrolment rate of 95.2% (40/42). Thirty-one participants (i.e., 77.5%) completed the intervention. Of those who completed the trial, the overall average attendance was reported as 85.2%; 84.7% in the TCTB group and 85.7% in the Tai Chi only group. A total of 58.3% of participants (*n* = 21) could independently practice the TCTB or Tai Chi exercise motions at the end of the learning stage. There were no missing data except for the nine participants who withdrew during the intervention. No adverse events or effects were reported, and all participants were satisfied with the 12-week exercise intervention. Results support the feasibility of a large-scale randomized controlled trial to explore the efficacy of a TCTB program for improving health in older sedentary office workers.

## 1. Introduction

An increasing number of older adults are participating in the labor workforce, with almost 64% of the Australian population aged between 55 and 64 years remaining in the labor workforce in 2014 [1]. Older adults experience a gradual reduction in physical function due to aging, physical inactivity, and a prolonged sitting time [2]. Consequently, older workers can also experience physical impairments (e.g., poor muscle strength and decreased endurance) [3]. Furthermore, heavy workloads, high job demand, and a lack of work support are found to be associated with work-related stress, depression, and anxiety [4]. Older workers, especially sedentary office workers, also report chronic pain such as low back pain, neck pain, and shoulder pain, which can be caused by prolonged sitting (e.g., at a computer) or limited physical activity [5].

Given the work-related physical impairments, psychological issues, and pain experienced among older office workers, researchers have examined several exercise interventions to improve health and well-being in the workplace (e.g., running, aerobic weight-bearing exercise) [6,7]. However, Parry and Coenen [8] found that it may be difficult for older office workers to partake in regular outdoor exercise as part of a workplace intervention due to the nature of their work. Tai Chi exercise and resistance training, which require a small space to practice, are suggested as appropriate exercises for older office workers as these two exercise programs can be easily performed within their workplace [9]. Tai Chi or resistance training have also been found to have the potential to improve physical fitness and psychological well-being of older adults as well as to reduce pain [10,11,12,13]. Given the existing body of evidence of Tai Chi or resistance training benefits in isolation, combining resistance training with Tai Chi might more effectively address physical fitness and psychological well-being compared with Tai Chi exercise only in older sedentary office workers.

Tai Chi, combined with resistance training, has recently been used to improve muscle strength, balance, and endurance of older adults [14,15]. However, these studies used Tai Chi on its own as part of the exercise program, followed by resistance training. Further to this, our published systematic review found that there was no available study that examined the potential effects of Tai Chi performed with resistance training on psychological well-being and pain in older adults or older office workers [16]. There is also no evidence for researchers to apply this new combined exercise approach (i.e., Tai Chi combined with Thera-band training, TCTB) to improve the health of older office workers. Given these limitations, we developed a TCTB program and conducted a 12-week pilot RCT to evaluate the feasibility and preliminary efficacy of TCTB to improve health and well-being in older sedentary office workers. Details of the study design and efficacy results from the study are reported in our published paper where the 12-week TCTB program was found to improve lower limb and right upper limb strength in older sedentary office workers [17]. However, there was a lack of feasibility outcomes about the study recruitment, acceptability of the study intervention as well as the participants’ satisfaction with the study intervention in the published paper. Thus, this paper focuses on the feasibility of the TCTB program (i.e., recruitment and enrolment, retention, adherence, learning and data collection as well as participants’ evaluation).

## 2. Materials and Methods 

### 2.1. Design 

The feasibility of the TCTB program protocol was assessed using a pilot randomized controlled trial (RCT) with two groups (i.e., TCTB group and Tai Chi group) that were conducted at a university in South East Queensland, Australia. Ethics approval for this study was received from the University Human Research Ethics Committee (GU-HREC Approval Number 2016/872). The study was registered with the Australian New Zealand Clinical Trials Register (ACTRN 12617001628336).

### 2.2. Participants and Recruitment

Participants were older office workers (i.e., administrative and academic staff) aged over 55 years, could walk independently, and were engaged in less than 60 min of accumulated moderate intensity physical activity (e.g., brisk walking, bicycling, and yoga) per week, which was self-assessed by potential participants. Exclusion criteria included significant vision impairment, participation in Tai Chi or Thera-band exercise of any kind in the last year, and a lack of ability to commit to Tai Chi exercise for 45 min three times per week. We aimed to enroll 40 participants within one month. The sample size of 40 participants for this study was considered adequate for a pilot RCT study for standardized effect sizes that range from small to medium [18]. 

Follow up to recruitment emails were sent one week after initial recruitment contact and then every three days for two weeks to remind potentially interested participants who did not provide signed consent. Consenting participants who were interested in taking part in the study were then screened according to the study inclusion and exclusion criteria, and the Physical Activity Readiness Questionnaire (PAR-Q). Participants who did not meet the criteria were informed by email that they were not eligible to partake in the study.

### 2.3. Exercise Intervention 

**Figure 1:**
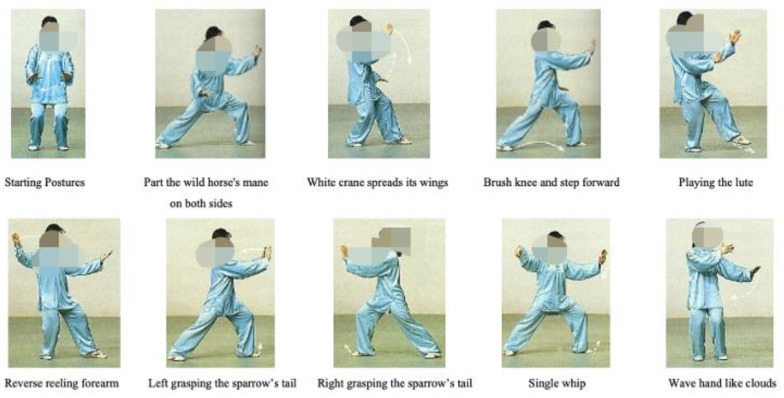
The first 10 movements of the 24-simplified Tai Chi forms.

The first 10 Tai Chi movements of the 24-simplified Tai Chi form [19] were adopted for both the TCTB group and the Tai Chi exercise group. Seven of the first 10 movements of the 24-simplified Tai Chi forms aim for arm separation and require resistance of the Thera-bands (i.e., this is described as “Part the wild horse’s mane on both sides”, “White crane spreads its wings”, “Brush knees and step forward”, “Reverse reeling forearm”, “Left/Right rasping the sparrow’s tail”, and the “Single whip”). The rest of the movements consist of “Starting postures”, “Play the lute”, and “Wave hands like clouds”. Pictorial depictions of the different movements are presented in Figure 1.

**Figure 2:**
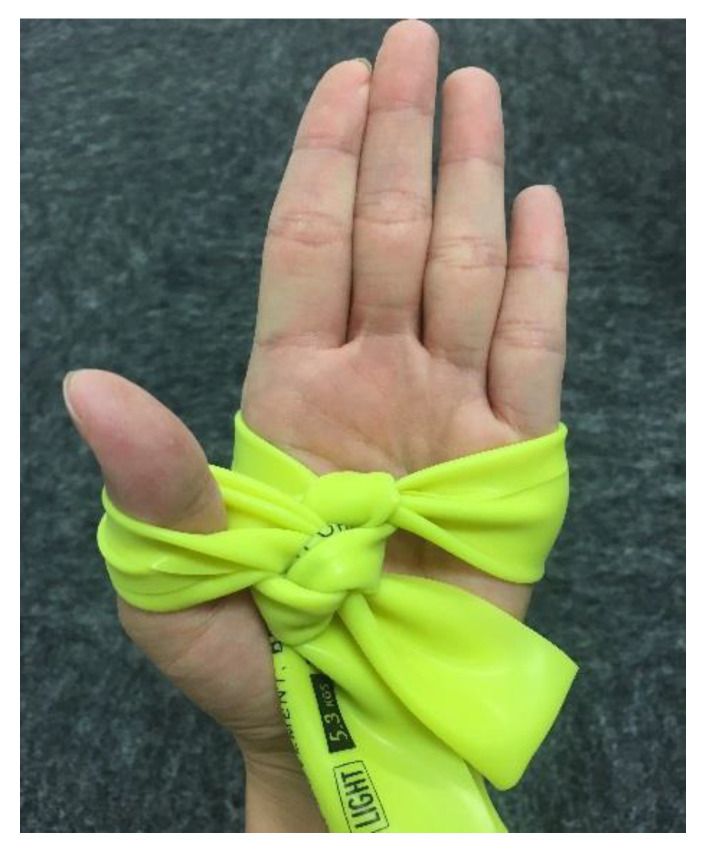
Thera-band knots.

**Figure 3:**
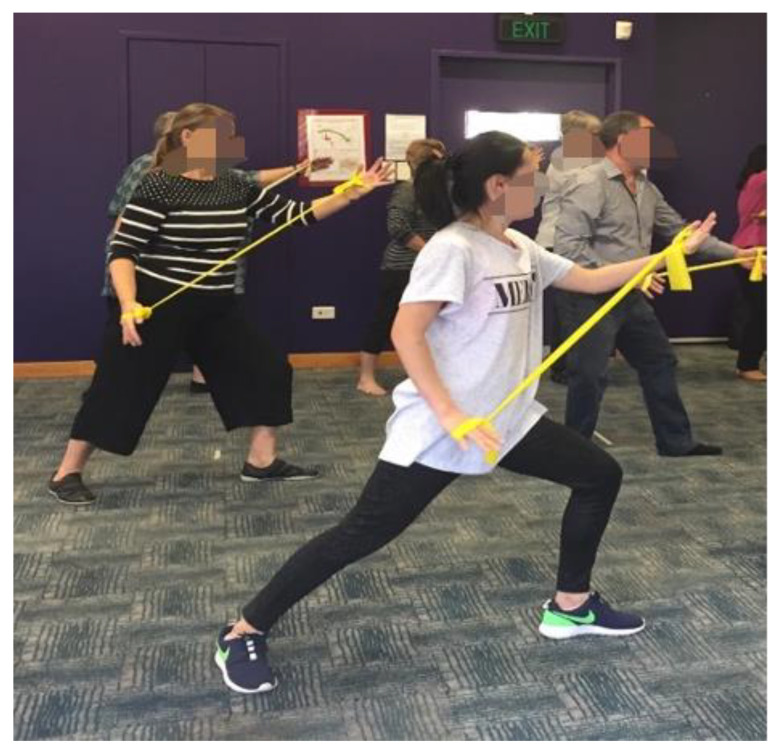
Tai Chi combined with Thera-band training.

A resistance band (Thera-band, The Hygenic Corporation, Akron, OH, USA) was used in the TCTB group. Each participant tied two knots on each side of the Thera-band, adjusting to the size of their palms and the thickness of their thumbs (see Figure 2). While practicing Tai Chi, participants used their two hands to wrap around the Thera-band (see Figure 3). The Thera-band color indicates the progressive resistance levels with tan reflecting the lowest intensity, followed by yellow, red, green, blue, black, silver, and gold. The resistance training was performed initially with tan Thera-bands for females, and yellow Thera-bands for males. After the learning stage, participants self-determined the appropriate resistance level of the Thera-band to use according to their own individual capacity.

All sessions were conducted in separate pre-booked seminar rooms at the University. Each session lasted for 45 min, three times per week. Each session was comprised of a 10-min warm up, 30-min Tai Chi or TCTB exercised practice, and 5-min cool down. The TCTB or Tai Chi exercise was taught incrementally over the first two-week learning stage (refer to Table 1). Participants were assessed at the end of the learning stage by the Tai Chi training instructor to ensure they could independently complete the TCTB or Tai Chi exercise before starting the 12-week intervention sessions. 

Clear instructions and demonstration of the exercises were provided by the Tai Chi training instructor, if needed, during the first three weeks of the 12-week intervention period (refer to Table 2). From week four onward, a video showing the Tai Chi exercise motions was shown on the session room’s screen. Relaxing Tai Chi background music was played on a mobile phone or the session room’s computer throughout the 12-week intervention. 

### 2.4. Data Collection and Analysis

Study feasibility was examined through recruitment and enrolment rates, participants’ retention and adherence, learning and data collection, and participants’ evaluation of the intervention. Data were analyzed descriptively (i.e., percentages, etc.) using IBM SPSS Statistics for Windows Version 23.0 where appropriate. 

Participant demographics were collected by the Tai Chi training instructor using an online survey before commencement of the exercise intervention. To track participant retention and adherence, participant adherence was recorded by the Tai Chi training instructor using the total number of exercise sessions attended by participants. Participants’ learning was assessed by the Tai Chi training instructor at the end of the 2-week learning stage. Participants who could complete the TCTB or Tai Chi exercise without input from the Tai Chi training instructor were considered to be able to practice independently in this study. The appropriateness of data collection was assessed using the total number of eligible and completed data collected at the various timepoints (i.e., baseline, week 6, and week 12). Although there were no qualitative interview data collected in this study, the Tai Chi training instructor’s observation notes during the study provided important information regarding the participants’ perceptions toward the study intervention. Participants’ oral and written evaluations also included valuable information relating to the participants’ acceptability of the study. Essential information from these evaluations was summarized and reported.

## 3. Results

The participants’ flowchart and the baseline characteristics of the participants are presented in our published paper [17]. 

### 3.1. Recruitment and Enrolment

Of the 2020 older office workers employed by the university, 42 participants were recruited over two rounds of recruitment lasting nearly 20 weeks, yielding a recruitment rate of 2.1 participants per week. This represented a low response rate of 2.0%. Of these consenting participants, 28 were recruited by email invitations and posters, five of them signed the consent form after being individually invited, three of them expressed their interest following their colleagues’ promotion, and six of them signed the consent form following promotion from the University Wellness Program. The primary barriers to join this study were cited as existing workload and scheduled family activities. Following assessment, one person was excluded as a result of engaging in more than 60 min of moderate physical activity per week. Another person declined to complete the baseline assessments for an unknown reason and was therefore excluded, leaving a high enrolment rate of 95.2% (i.e., 40 out of 42 participants). 

### 3.2. Retention and Adherence

Of the 40 participants enrolled in the study, nine withdrew their participation during the study, with five from the TCTB program group and four from the Tai Chi group, reflecting a final participant retention and completion rate of 77.5%. Four participants dropped out after the two-week learning stage, with reasons including confusion over the session room locations (*n* = 1), knee pain unrelated to the intervention (*n* = 2), and undeclared (*n* = 1). During the intervention stage, two participants dropped out at the end of week 2 citing back pain unrelated to the intervention (*n* = 1) and the flu (*n* = 1). One participant dropped out at the end of week three with no reason provided. A further two participants dropped out at the end of weeks three and four due to competing commitments. When attrition was taken into consideration, the overall average attendance rate for the 31 participants was 85.2%, with 84.7% in the TCTB group and 85.7% in the Tai Chi group. Reasons for non-attendance in both groups included workload, family issues, and competing commitments. 

### 3.3. Learning and Data Collection 

At the end of the learning stage, four participants withdrew their participation, leaving 36 participants. Records of the participants’ learning revealed that almost half of the participants (58.3%, 21/36) could practice the TCTB or Tai Chi exercise motions independently. The rest of the participants could also execute the movements with guidance from the Tai Chi training instructor during the first three weeks of the intervention. However, they were able to practice the TCTB or Tai Chi exercises independently by the end of week 3. At the start of week 4, the Tai Chi video was introduced, and all of the participants found that it was easy to keep up with the pace of the exercise motions in the video. 

All forty eligible participants completed the baseline demographic assessment. Following attrition, 31 remaining participants completed the outcome assessment at the end of week 6 and week 12, with no missing data recorded. All participants completed further psychological and pain assessment via an online survey within three days of receiving the reminder emails. Seven and six participants were late to the physical fitness assessment session at week 6 and week 12, respectively, the main reasons they gave were time conflicts and change of job location. 

### 3.4. Participants’ Evaluation

No adverse events or effects were reported during the 12-week exercise intervention. All participants (P) in both the TCTB and Tai Chi exercise group enjoyed the exercise program (*“I am happy to have been part of the data”—P5 from the Tai Chi group; “I certainly enjoyed the classes that I was able to attend and enjoyed meeting all the other Taichiers”—P1 from the Tai Chi group; “We had so much fun learning this new activity”—P3 and P6 from the TCTB group*). They also felt that their mental health improved after completing both the TCTB and Tai Chi exercises (*“I felt Tai Chi has been good for my mood.....Tai Chi reduced my feeling of stress and depression”—P8 from the TCTB group; “I felt good, my stress has been reduced”—P9 from the Tai Chi group*), with more participants in the TCTB group reporting improved muscle strength (*“Yes, my arms got stronger……as well as my legs—P3 from the TCTB group*). Thus, the majority of the participants (i.e., 12 out of 16) in the Tai Chi group expressed a desire to learn and practice the TCTB exercises after the program (*“The Tai Chi with Thera-bands certainly stood out, I would also love to learn a little of the band exercise”—P7 from the Tai Chi group*). The trainer was highly regarded by all participants who felt that she was patient and helpful in teaching the exercise motions (*“She is a very kind special person”—P8 from the Tai Chi group; “She is amazing, and I would love to introduce other people to her exercise program”—P3 from the TCTB group*). Of those who withdrew from the study, several emailed to express their enjoyment of the TCTB or Tai Chi exercises. They also expressed their regret in having to withdraw from the study (*“I really liked it and have wanted to try it for some time now, but I do not think it is for me”—P11 from the TCTB group; “It is regretful, but I will have to withdraw from your research study due to the uncertain nature and time frames of my role at Griffith”—P13 from the Tai Chi group*). Most importantly, of those who withdrew due to reported knee or back pain (n = 3), their reported pain was not as a result of participating in the study (*“Just to be clear, doing Tai Chi last week did not hurt my back, it is something I have had for years”—P2 from the TCTB group*). All remaining 31 participants expressed their interest and desire to continue with the Tai Chi or the TCTB exercises after the intervention (*“I do keep doing the exercise now and would love to rejoin a class if you are thinking of doing some more”—P9 from the Tai Chi group; “I am still practicing Tai Chi* (*12 forms with band*) *each weekdays morning, seven cycles each time-taking around 26 min”—P14 from the TCTB group*).

## 4. Discussion

In this pilot study, we sought to determine the feasibility of a TCTB exercise program for improving health and well-being in older sedentary office workers. Except for the low recruitment and response rate, and the slow progress of participant recruitment, the findings suggest that a 12-week TCTB intervention designed to improve physical fitness and psychological well-being and reduce pain can be feasibly delivered to older sedentary office workers. 

The recruitment and response rate of this pilot study seemed lower than in other physical activity studies in smaller health or research agencies [20,21]. The challenges of recruitment could partially be due to the different study setting. A university is much larger than a health agency, which made it difficult to gather all university staff in one place for recruitment. Only a small proportion of staff might match the target population of sedentary office workers among the 2020 older employees contacted. The use of university email as the primary method to recruit participants also makes it challenging to determine the overall success of the email recruitment as advertisement reach (i.e., open email rate) is unknown [22]. However, our pilot study was promoted via an email sent to all staff as part of the university wellness program and supported by Chau and Daley [20], who demonstrated that treating a study as part of a workplace wellness program might be an effective way to recruit an adequate number of participants within a short period. On the other hand, the high enrolment rate might be attributed to the clear and detailed information provided in the recruitment materials (i.e., information sheet and recruitment flyer) including the purpose and background of the study, participation eligibility criteria, potential risks and benefits of the study, required level of involvement in the study as well as the inclusion of appropriate Tai Chi and Thera-band training photos. Overall, future studies may consider seeking collaboration with workplace wellness programs and preparing clear and detailed information sheets for participants to facilitate the recruitment and enrolment process. 

Although the participants’ retention and completion rate can be found from the participants’ flowchart in our published paper, the reasons for the participants’ withdrawal were not reported. The high participants’ retention and adherence rate suggest that the TCTB program is a feasible intervention in older sedentary office workers. For participants who were unable to continue with the study, their reported reasons for withdrawal were similar to those reported in other studies including time conflicts and existing health problems [14,23]. Nevertheless, there was a unique reason for confusion cited by one participant who withdrew from the pilot study. The confusion was due to the rooms where the sessions were conducted. It was difficult to consistently reserve one single specific room for the implementation of all sessions in a large university setting throughout the study period. Hence, sessions were conducted across several different rooms that caused some confusion during the intervention period. It is, therefore, essential to consider this factor when planning for physical activity interventions in future studies. 

Data collection for the pilot study was successful when compared to those in previous studies concerning workplace health promotion in sedentary workers and university employees where only a data collection rate ranging from 59% to 86% was reported [24,25]. Potential reasons for successful data collection might include the use of an online survey design with follow-up email reminders and the provision of incentives (i.e., in this case, participants were given a small towel and an exercise band). The successful data collection suggests that follow-up emails and incentives may increase the data collection process. However, the small sample size might also contribute to the successful data collection, which is a weakness of this study. Additionally, participants’ positive feedback and interest to continue the TCTB program or Tai Chi exercises after the intervention also support that the TCTB program may be a possible treatment for older sedentary office workers. Future studies could also conduct an evaluation of the participants’ experience with the TCTB program via a Likert scale questionnaire.

## 5. Conclusions

This pilot study highlights some of the challenges in recruiting older office workers from a university setting. However, the excellent participant retention and adherence, and successful data collection suggest that a larger trial may be feasible. The participants’ informal feedback and comments received throughout and following the intervention provide further insight into the feasibility of the intervention and its outcomes. Results and experiences gained from this pilot study need to be considered when planning for a future study to assess the effectiveness of the TCTB program in increasing physical fitness, improving psychological well-being, and reducing pain for older sedentary office workers. 

## Figures and Tables

**Table 1:** Learning stage exercise program session schedule.

Week/Session	Warm-Up	Learning	Cool-Down
Week 1-Session 1	Movements 1–3	Tai Chi movements 1–3	Movements 1–3
Week 1-Session 2	Movements 1–5	Tai Chi movements 4–6	Movements 1–3
Week 1-Session 3	Movements 1–5	Tai Chi movements 7–10	Movements 1–3
Week 2-Session 1	Movements 1–5	Tai Chi movements 1–10 holding Thera-bands	Movements 1–3
Week 2-Session 2	Movements 1–5	Tai Chi movements 1–10 holding Thera-bands	Movements 1–3
Week 2-Session 3	Movements 1–5 assessment	Tai Chi movements 1–10 holding Thera-bands assessment	Movements 1–3 assessment

**Table 2:** The 12-week intervention practice stage with background music.

Week/Session	Warm-Up	Learning	Recapping	Cool-Down
Week 1-All sessions	Movements 1–5	Instruction if needed	TCTB 1–10	Movements 1–3
Week 2-All sessions	Movements 1–5	Instruction if needed	TCTB 1–10	Movements 1–3
Week 3-All sessions	Movements 1–5	Instruction if needed	TCTB 1–10	Movements 1–3
Week 4-All sessions (with video)	Movements 1–5	-	TCTB 1–10	Movements 1–3
Week 5-All sessions (with video)	Movements 1–5	-	TCTB 1–10	Movements 1–3
Week 6-Sessions 1–2 (with video)	Movements 1–5	-	TCTB 1–10	Movements 1–3
Week 6-Session 3	6-week measurement assessment			
Week 7-All sessions (with video)	Movements 1–5	-	TCTB 1–10	Movements 1–3
Week 8-All sessions (with video)	Movements 1–5	-	TCTB 1–10	Movements 1–3
Week 9-All sessions (with video)	Movements 1–5	-	TCTB 1–10	Movements 1–3
Week 10-All sessions (with video)	Movements 1–5	-	TCTB 1–10	Movements 1–3
Week 11-All sessions (with video)	Movements 1–5	-	TCTB 1–10	Movements 1–3
Week 12-Sessions 1–2 (with video)	Movements 1–5	-	TCTB 1–10	Movements 1–3
Week 12-Session 3	12-week measurement assessment			

TCTB, Tai Chi combined with Thera-band.
